# Migraine Symptoms Improvement During the COVID-19 Lockdown in a Cohort of Children and Adolescents

**DOI:** 10.3389/fneur.2020.579047

**Published:** 2020-10-08

**Authors:** Gianfranco Dallavalle, Elena Pezzotti, Livio Provenzi, Federico Toni, Adriana Carpani, Renato Borgatti

**Affiliations:** ^1^Department of Brain and Behavioral Sciences, University of Pavia, Pavia, Italy; ^2^Child Neurology and Psychiatry Unit, IRCCS Mondino Foundation, Pavia, Italy

**Keywords:** COVID-19, children, adolescents, migraine, headache, stress, anxiety

## Abstract

**Background:** Pediatric migraine is among the most common primary or comorbid neurologic disorders in children. Psychological stressors are widely acknowledged as potential triggers involved in recurring episodes of pediatric migraine. As the COVID-19 emergency may have affected the levels of stress perceived by children and adolescents with migraine, the present study was aimed to understand the effect of COVID-19 emergency on symptoms intensity and frequency in pediatric patients.

**Methods:** A cohort of 142 child and adolescent patients with a diagnosis of migraine was enrolled at the Child Neurology and Psychiatry Unit of the IRCCS Mondino Foundation in Pavia (Italy). Socio-demographic and clinical characteristics were obtained from medical records. An on-line survey was used to collect information on COVID-19 exposure, stress response to the lockdown period, anxious symptoms during COVID-19 emergency, as well as migraine symptoms intensity and frequency before and during the lockdown.

**Results:** The great majority were outpatients (*n* = 125, 88.0%), 52 (36.6%) had migraine with aura, whereas, 90 (63.4%) had migraine without aura. All the patients reporting worsening symptoms progression before COVID-19, had reduced intensity during the lockdown (χ^2^ = 31.05, *p* < 0.0001). Symptoms frequency reduction was observed in 50% of patients presenting worsening symptoms before the lockdown, 45% of those who were stable, and 12% of those who were already improving. All patients who had resolved symptoms before COVID-19 were stable during the lockdown (χ^2^ = 38.66, *p* < 0.0001). Anxious symptomatology was significantly associated with greater migraine symptoms frequency (χ^2^ = 19.69, *p* < 0.001). Repeating the analysis separately for individuals with and without aura did not affect the findings and significant associations were confirmed for both the patients' subgroups.

**Discussion:** A significant reduction of migraine symptoms intensity and frequency was observed in pediatric patients during the COVID-19 lockdown phase in northern Italy. The improvement in both intensity and frequency of the migraine symptoms was especially significant in patients who were stable or worsening before the lockdown. The reduction of symptoms severity during a period of reduced environmental challenges and pressures further highlights the need of providing effective training in stress regulation and coping for these patients.

≪*Some patients I could help with drugs, and some with the magic of attention and interest…it now became apparent to me that many migraine attacks were drenched in emotional significance*≫.

[Oliver Sacks – Migraine, 1970]

## Introduction

During the 1st months of 2020, Northern Italy has been the hotspot of the coronavirus disease of 2019 (COVID-19) outbreak in Europe (1). The adopted mitigation and containment actions included physical distancing strategies that indirectly resulted in the lockdown of schools and changes in daily habits. In this scenario, citizens may have been exposed to high levels of stress and anxiety (2). The mental health impact of this unprecedented healthcare emergency might be especially significant for children who already were suffering from physical and/or psychosomatic conditions, as it is the case of pediatric population with migraine (3, 4). Pediatric patients with migraine have been previously reported to be especially vulnerable to stressful and anxious encounters (3, 4). Thus, these patients represent a specific at-risk population that should be monitored for COVID-19-related effects on their health and symptoms progression.

Pediatric migraine is among the most common primary or comorbid neurologic disorders in children, with prevalence ranging from 3% in preschool children to 23% in adolescents (5, 6). Migraine may be generally considered as a disorder of psychobiology adaptation where genetic predisposition plays a critical role together with internal and external sources of environmental influence, including psycho-social and psycho-emotional challenges, hormonal dysregulation, dietary and other factors (3). A complex mix of factors is plausibly involved in setting the risk for pediatric migraine, including neurogenic inflammation, excitatory/inhibitory balance, genetic background and disturbed energy metabolism (7–9). Psychosomatic contributions have recently supported by neuroimaging studies as the default mode network appears to play a critical role in mediating the effects of environmental stressors and coping strategies on the origin and emergence of migraine symptoms (7).

Psychological stressors are widely acknowledged as potential triggers involved in recurring episodes of pediatric migraine (10, 11). Stressful, challenging and emotionally overwhelming experiences in school or educational environments may contribute to the overreaction of the central nervous system to environmental requests that are perceived as too intense by the individual, increasing the risk of headache and migraine (12). In large cohort studies, children with frequent and more intense migraine symptoms also report higher levels of school, family and/or peer-relational stress compared to headache free counterparts (13–15).

There is evidence of COVID-19 pandemic effects' on the psychological and physical well-being of children and adults in the general population (16–19). Recent research conducted in Italy reported that, during the COVID-19 quarantine, subjects with migraine had fewer migraine attacks and lesser pain as well as moderate levels of depression (20). Nonetheless, no information is available for what pertains the health of at-risk children and adolescents with pediatric migraine. In the present study we report the results of a survey conducted at a tertiary level neurological hospital in northern Italy. The survey was aimed to collect evidence on the impact of COVID-19 lockdown phase on the frequency and intensity of migraine symptoms among children and adolescents.

## Methods

From March to April 2020, a cohort of 142 child and adolescent patients with a diagnosis of migraine was enrolled at the Child Neurology and Psychiatry Unit of the IRCCS Mondino Foundation in Pavia (Italy). This hospital receives families for inpatient and outpatient care from Lombardy and other Italian regions. Patients were consecutively enrolled provided that parents could speak and understand Italian language. Patients were included if they did not present any comorbidity (e.g., psychomotor delay, neuromuscular diseases, epileptic disorders, cerebral palsy). The parents were asked to respond to an *ad-hoc* on-line survey targeting the exposure to COVID-19, anxious symptoms during COVID-19 emergency, as well as migraine symptoms intensity and frequency before and during the lockdown (Table 1). Participation was anonymous and voluntary. Consent of parents was obtained according to local procedures.

**Table 1:** Survey structure and items description.

**Item N**	**Item text**	**Response option**
1	I live in a COVID-19 outbreak area	0, no; 1, yes
2	At least one family member was positive to COVID-19	0, no; 1, yes
3	At least one family member has to travel to COVID-19 areas for job duties	0, no; 1, yes
4	School activities were continuing in remote	0, no; 1, yes
5	Sport/leisure activities were suspended	0, no; 1, yes
6	My child anxious symptoms changed during the lockdown	0, worsening; 1, stable; 2, improving
7	The intensity of migraine symptoms was changing before the lockdown	0, worsening; 1, stable; 2, improving; 3, resolution
8	The intensity of migraine symptoms changed during the lockdown	0, worsening; 1, stable; 2, improving; 3, resolution
9	The frequency of migraine symptoms was changing before the lockdown	0, worsening; 1, stable; 2, improving; 3, resolution
10	The frequency of migraine symptoms changed during the lockdown	0, worsening; 1, stable; 2, improving; 3, resolution

Sociodemographic (sex, age, and ethnicity) and clinical variables (i.e., patient status, presence of aura) were obtained from medical charts. Separate χ^2^ tests were used to test changes in migraine symptoms intensity and frequency from before COVID-19 to the lockdown period. A second set of χ^2^ tests was used to test the association of anxious symptomatology with both intensity and frequency of migraine symptoms progression during the lockdown. Statistic tests were considered significant if *p* < 0.05. All *p*-values were 2-tailed.

## Results

The mean age of participants (78 females, 54.9%) was 15.04 years (range [5, 21], *SD* = 3.23). The great majority were outpatients (*n* = 125, 88.0%), 52 (36.6%) had migraine with aura, whereas 90 (63.4%) had migraine without aura. Among patients with aura, 34 had visual aura (65.4%), three patients had brainstem aura (5.8%), one patient had sensory aura (1.9%) and one patient had aura with motor disturbances (1.9%). Moreover, 13 patients (25.0%) reported mixed aura including different patterns of visual, sensory, motor, language, and brainstem disturbances. Fifty-two patients (36.6%) were living in the first Italian geographical hotspot of COVID-19 spread. Twelve patients (8.5%) had at least one relative who was positive to the virus. Sixty patients (42.3%) had at least one parent who needed to travel to a COVID-19 area for job duties. School activities were continuing in remote for 130 patients (91.6%) and sport/leisure activities were suspended for 125 patients (88.0%) at the time of the survey.

**Figure 1:**
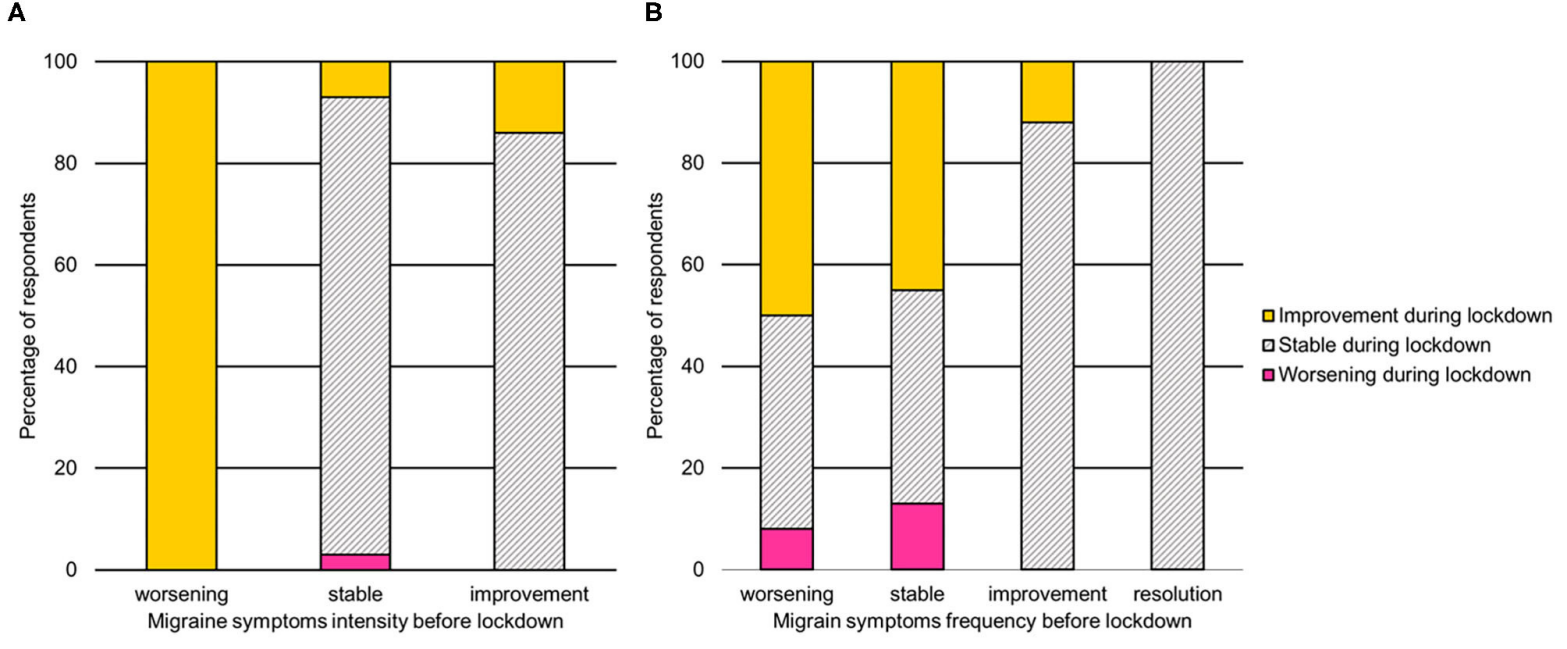
Association between migraine symptoms intensity **(A)** and frequency **(B)** before and during the lockdown. Note. The symptoms severity before lockdown is reported on the x-axis, whereas the symptoms severity during the lockdown is reported using color gradients.

The association between symptoms intensity and frequency before and during the lockdown is reported in Figures 1A,B. Migraine symptoms intensity worsened in four patients (2.8%) and improved in 13 cases (9.2%) during the lockdown. All the patients reporting worsening symptoms progression before COVID-19, had reduced intensity during the lockdown (χ^2^ = 31.05, *p* < 0.0001). Frequency of migraine symptoms worsened in nine patients (6.3%) and improved in 40 cases (28.2%). Symptoms frequency reduction was observed in 50% of patients presenting worsening symptoms before the lockdown, 45% of those who were stable, and 12% of those who were already improving. All patients who had resolved symptoms before COVID-19 were stable during the lockdown (χ^2^ = 38.66, *p* < 0.0001).

During the lockdown, anxiety symptoms worsened in 37 patients (26.1%), were stable in 104 patients (73.2%), and improved only in one patient. Anxious symptomatology was significantly associated with greater migraine symptoms frequency (χ^2^ = 19.69, *p* < 0.001), but not intensity (χ^2^ = 1.24, *p* = 0.54). Repeating the analysis separately for individuals with and without aura did not affect the findings and significant associations were confirmed for both the patients' subgroups.

## Discussion

This study highlighted a significant reduction of the intensity and frequency of migraine symptoms in the present cohort of children and adolescents during the COVID-19 lockdown phase in northern Italy. The improvement in both intensity and frequency of the migraine symptoms was especially significant in patients who were stable or worsening before the lockdown. Additionally, patients who were already improving before the healthcare emergency, reported a stable clinical picture of migraine symptoms severity in terms of intensity and frequency during the lockdown.

This finding is only apparently counterintuitive. For children and adolescents with recurring and worsening presentations of migraine, without any other comorbidity, the lockdown coincided with a dramatic reduction of potential stress-factors that may act as triggers for symptoms intensity and frequency. We hypothesized that the suspension of school and sport activities, the limitation of physical contacts with peers and the overall reduction of environmental requests may had potentially resulted in a fail-safe effect on the daily psychological stress usually lived by these patients before the lockdown. Psychological stressors are widely acknowledged as potential triggers involved in recurring episodes of pediatric migraine (10, 11). As such, the COVID-19 lockdown may have produced unexpected, yet relevant relief from migraine symptoms for these patients. Previous research suggested that stressful psychological experiences in school and/or family may widely affect pediatric migraine symptoms (12). From this point of view, this finding further suggests that pediatric migraine may have a relevant – yet partial – psychosomatic nature (10), and dramatic situations such as a sudden change in daily habits can lead to unexpected improvements in the clinical picture.

Additionally, clinical worsening of migraine frequency was only observed in those patients reporting higher anxiety during the lockdown phase. The comorbidity of anxious symptomatology with migraine is well-documented in children and adolescents (21, 22). Additionally, previous research reported on the significant association between migraine frequency and mood disorders (23). Moreover, anxious symptomatology is one of the psychosocial and affective factors involved in pediatric migraine onset and chronicity (21, 24) and similar mechanisms have been theorized to be in place for both anxiety and chronic pain (25, 26). This finding is of critical importance for at least two major reasons. First, the worsening of symptoms in patients who also reacted to the lockdown phase with increasing anxiety is reminiscent of the central involvement of psychological distress in the recurrence of headache symptoms in these children and adolescents. As anxiety symptoms were rated by parents, a careful exploration of anxious symptoms progression in daily life should be always considered by healthcare providers and may be conducted in partnership with the patient and the family. Second, a relatively small – yet clinically compelling – percentage of patients (i.e., 37 out of 142; 26%) reported anxiety symptoms worsening during the lockdown phase. This means that approximately one out of four patients with pediatric migraine may have experienced a relevant reduction of their mental health and psychological well-being during the COVID-19 emergency. As such, young patients with migraine should be considered as a specific vulnerable population that needs specialized and multi-professional attention during and after the epidemic, or major stressing events.

## Limitations

Although this study only included the enrollment of patients from a single hospital, it should be highlighted that the IRCCS Mondino Foundation receives patients and families from different regions of the Italian territory. Moreover, this survey only included parent-reported data and the indirect nature of this survey did not allow the collection of observational data on the quality of life experienced by patients and their parents during the lockdown. The lack of standardized and quantitative measures of pain intensity and/or frequency is another limitation to this study. Similarly, internalizing behaviors may affect pain perception in children (27) and were not assessed in this study. Finally, socio-demographic and socio-economic confounders have been previously associated with the incidence and severity of migraine (28, 29) and their role in affecting patients' symptoms cannot be completely ruled out in the present survey.

## Conclusions

Taken together, these findings suggest that the COVID-19 lockdown phase may had resulted in an unexpected relieving improvement of migraine symptoms' frequency and intensity in pediatric patients. It is well-known that daily sources of psychological stress may act as triggers of migraine symptoms in children and adolescents (30). One can speculate that this unexpected improvements in migraine symptoms could be – at least partially – related to a reduction in external or internal demands for high performance in daily social settings, such as school and sport or leisure activities (10). On a theoretical level, these findings further confirms the role played by psychosocial factors in the onset, progression and stabilization of migraine symptoms in children and adolescents (10). Moreover, as psychological stress inherent to academic and social life can be a prominent factor linked with migraine symptoms severity, this study also underlines the need of promoting interventions aimed at improving stress resilience and coping in pediatric patients' with migraine (31). For example, focusing on psychological and environmental aspects of child and adolescents' migraine in a multidisciplinary, continuous and integrated healthcare approach is warranted to improve patients' outcomes and quality of life (32, 33).

## Data Availability Statement

The raw data supporting the conclusions of this article will be made available by the authors, upon reasonable request.

## Ethics Statement

The study was reviewed and approved by Ethics Committee Pavia. Written informed consent to participate in this study was provided by the participants' legal guardian/next of kin.

## Author Contributions

RB: has full access to all of the data in the study and takes responsibility for the integrity of the data and the accuracy of the data analysis. EP and GD: concept and design. AC, GD, EP, and FT: acquisition of data. LP: data analysis and drafting of the manuscript. RB: supervision. All authors interpretation of data and critical revision of the manuscript for important intellectual content.

## Conflict of Interest

The authors declare that the research was conducted in the absence of any commercial or financial relationships that could be construed as a potential conflict of interest.
